# The role of the interleukin-36 axis in generalized pustular psoriasis: a review of the mechanism of action of spesolimab

**DOI:** 10.3389/fimmu.2023.1292941

**Published:** 2023-11-21

**Authors:** Jason E. Hawkes, Sudha Visvanathan, James G. Krueger

**Affiliations:** ^1^ Integrative Skin Science and Research and Pacific Skin Institute, Sacramento, CA, United States; ^2^ Translational Medicine and Clinical Pharmacology, Boehringer Ingelheim Pharmaceuticals, Inc., Ridgefield, CT, United States; ^3^ Laboratory for Investigative Dermatology, The Rockefeller University, New York, NY, United States

**Keywords:** generalized pustular psoriasis, GPP, psoriasis, IL-36, IL-1, IL36RN, spesolimab

## Abstract

Generalized pustular psoriasis (GPP) is a rare, chronic, inflammatory skin disorder characterized by recurrent flares associated with skin erythema, desquamation, and widespread superficial sterile pustules, which may be severe (“lakes of pus”). Systemic symptoms are often present, including malaise, fever, and skin pain. In GPP, innate immune responses are driven by abnormal activation of the interleukin (IL)-36-chemokine-neutrophil axis and excessive neutrophil infiltration. This review highlights the IL-36 pathway in the context of the IL-1 superfamily and describes how unopposed IL-36 signaling can lead to the development of GPP. Targeted inhibition of the IL-36 receptor (IL-36R) is an attractive therapeutic strategy in the treatment of GPP, including flare prevention and sustained disease control. Spesolimab is a first-in-class, humanized, monoclonal antibody that binds specifically to the IL-36R and antagonizes IL-36 signaling. Spesolimab was approved by the US Food and Drug Administration in September 2022 to treat GPP flares in adults and was subsequently approved for GPP flare treatment in other countries across the world. Anti-IL-36R therapy, such as spesolimab, can mitigate flares and address flare prevention in GPP, presumably through rebalancing IL-36 signaling and modulating the pro-inflammatory response of the downstream effectors.

## Introduction

**Figure 1:**
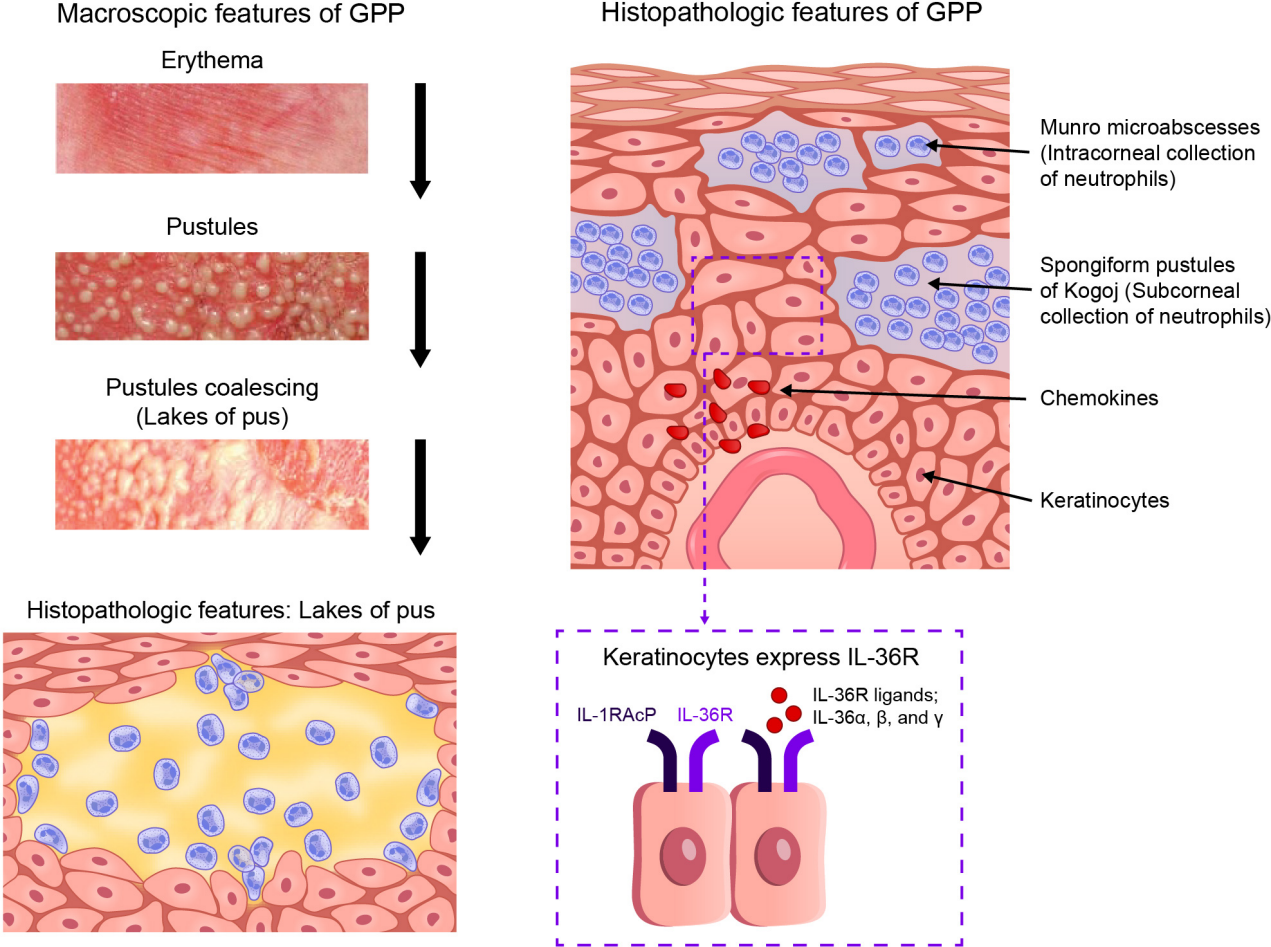
Key features of GPP. GPP, generalized pustular psoriasis; IL-1RAcP, interleukin-1 receptor accessory protein*; IL-36R, interleukin-36 receptor. (*Also known as IL-1R3).

Generalized pustular psoriasis (GPP) is a rare, chronic, inflammatory skin disorder, and is characterized by recurrent flares of erythema, desquamation, and the widespread eruption of superficial sterile pustules (1). In severe cases, the pustules may coalesce to form larger lesions known as “lakes of pus”. The key features of GPP are shown in **Figure 1**. Systemic symptoms often occur during episodic flares (1), including fever, malaise, and skin pain. Severe GPP flares often necessitate emergency or inpatient hospital care (2), due to the potential for complications, such as sepsis, heart failure, renal failure, and even death, if timely treatment is not provided (2, 3). The term “skin failure” was recently proposed to describe the potentially catastrophic endpoints of GPP (4). Estimates of GPP prevalence vary considerably in different regions of the world, ranging from approximately 2 to 120 cases per million persons (5–9). Reported mortality rates in patients with GPP are also variable, ranging from 0 to 3.3 deaths per 100 patient-years, with older studies (before 2000) stating higher rates than more recent studies (8). A 2021 study of Japanese patients with GPP who required hospitalization (N = 1516) reported a mortality rate of 4.2% (patient-year data were not available) (8, 10).

The clinical course of GPP is often heterogenous, and may present as relapsing (>1 episode within weeks, months, or years) or persistent disease (episode lasting >3 months) (1). GPP flares are often precipitated by a trigger, such as infection, emotional stress, pregnancy, hypocalcemia, or exposure to sunlight. A GPP flare may also be triggered by the withdrawal of systemic corticosteroids (11), or by exposure to a range of drugs, including lithium, antimalarials, ustekinumab, and some tumor necrosis factor (TNF) antagonists (12, 13).

Recent clinical, histological, and genetic data indicate that GPP is distinct from psoriasis vulgaris (PV, also called plaque psoriasis) (1, 14–16), and warrants separate diagnosis. While GPP can manifest concurrently with PV (1, 9, 17), GPP may occur in patients with no prior history of psoriatic disease (3). Abnormal activation of the interleukin (IL)-36-chemokine-neutrophil axis, dysregulation of innate immune responses, and ensuing excessive neutrophil infiltration are implicated in the pathogenesis of GPP (18); whereas, PV is an autoimmune disease characterized predominantly by IL-23/17 signaling and self-sustaining inflammatory cycles [positive feed-forward inflammatory response (19)] that lead to disordered proliferation and abnormal differentiation of keratinocytes (20).

The purpose of this review is to provide an overview of the IL-36 pathway in the context of the IL-1 superfamily and describe how unopposed IL-36 signaling can lead to the development of GPP. We will also discuss spesolimab, a novel first-in-class humanized monoclonal antibody that binds specifically to IL-36 receptor (IL-36R) and antagonizes IL-36 signaling. Spesolimab was approved by the US Food and Drug Administration (FDA) in September 2022 for the treatment of GPP flares in adults (21, 22), and was subsequently approved for GPP flare treatment in other countries across the world.

## IL-36 and the IL-1 superfamily

IL-36 cytokines belong to the IL-1 superfamily of cytokines (23–25). These are important regulators of the innate immune system, manifested by inflammation (26), and are essential for skin barrier function (27). IL-1 (α and β) was discovered first and, thus, is the best characterized of the 11 members of the IL-1 cytokine superfamily (25). IL-1 cytokines include receptor agonists (IL-1α, IL-1β, IL-18, IL-33, IL-36α, IL-36β, and IL-36γ), receptor antagonists (IL-1Ra, IL-36Ra, and IL-38), and an anti-inflammatory cytokine (IL-37) (23). Details of IL-1 family proteins and genes are presented in **Table 1** (23, 28).

**Table 1:** IL-1 family ligands, genes, and receptors (23, 28).

Sub-family	Cytokine ligand	Other name	Gene	Receptor	Activity
IL-1	IL-1α	IL-1F1	*IL1A*	IL-1R1	Pro-inflammatory
IL-1	IL-1β	IL-1F2	*IL1B*	IL1-R1	Pro-inflammatory
IL-1	IL-1Ra	IL-1F3	*IL1RN*	IL-1R1	Anti-inflammatory
IL-1	IL-33	IL-1F11	*IL33*	IL-1R4	Pro-inflammatory
IL-18	IL-18	IL-1F4	*IL18*	IL-1R5	Pro-inflammatory
IL-18	IL-37	IL-1F7	*IL1F7*	IL-1R5	Anti-inflammatory
IL-36	IL-36α	IL-1F6	*IL36A*	IL-1R6	Pro-inflammatory
IL-36	IL-36β	IL-1F8	*IL36B*	IL-1R6	Pro-inflammatory
IL-36	IL-36γ	IL-1F9	*IL36G*	IL-1R6	Pro-inflammatory
IL-36	IL-36Ra	IL-1F5	*IL36RN*	IL-1R6	Anti-inflammatory
IL-36	IL-38	IL-1F10	*IL1F10*	IL-1R6	Anti-inflammatory

**Figure 2:**
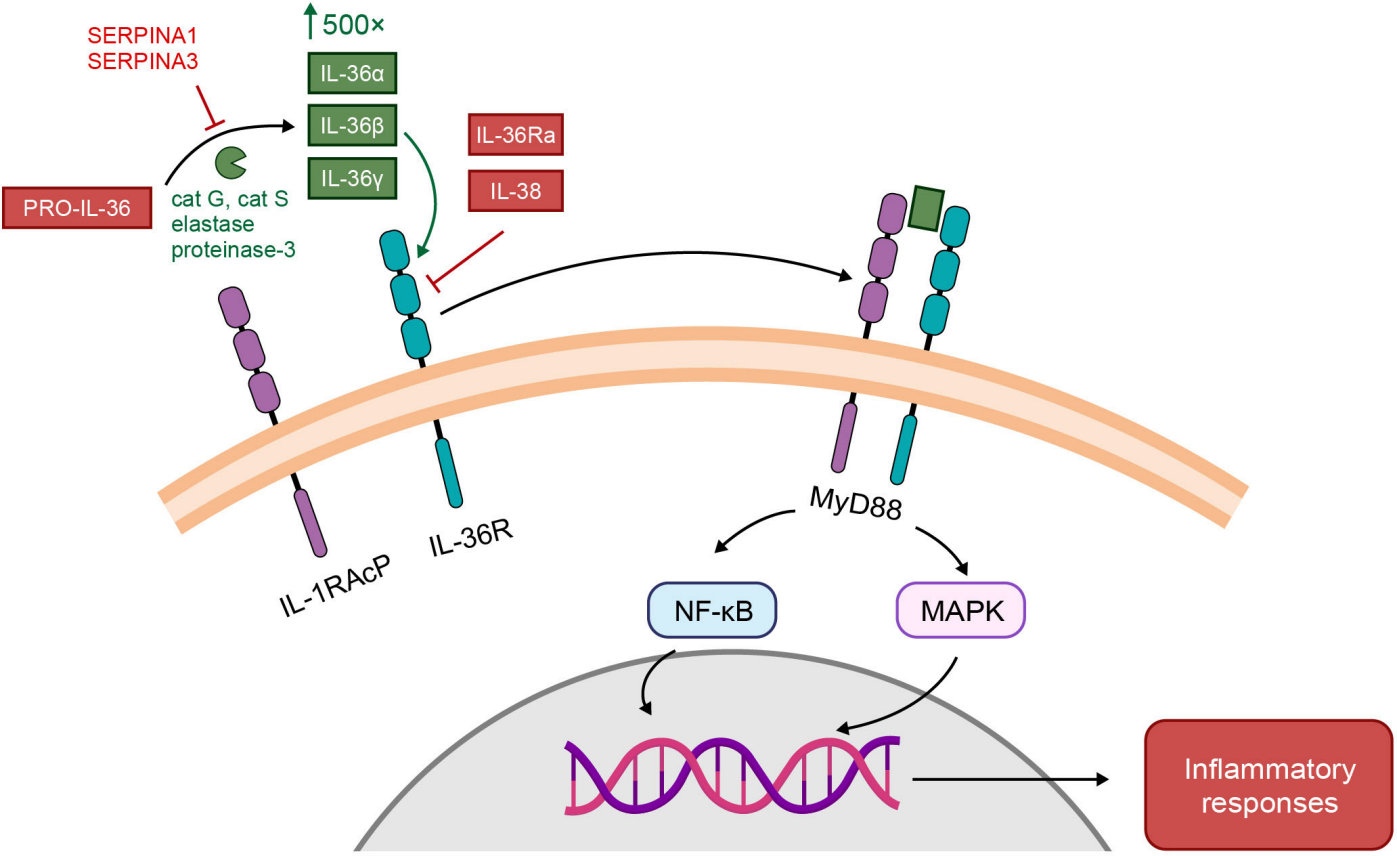
Receptor and signaling pathways activated by IL-1 and IL-36 (29). IL-36 activation pathway. IL-36 cytokines are secreted as low-activity precursors, pro-IL-36, which by the action of various proteases (cat G-cathepsin G; cat S-cathepsin S; elastase; proteinase-3) are cleaved into biologically active IL-36 agonists, IL-36α, IL-36β, and IL-36γ or antagonist IL-36Ra. IL-36 agonist processing increases their biologic activity by roughly 500-fold. IL-36 agonists form a binary complex with IL-36R, which recruits the IL-1 receptor accessory protein (IL-1RAcP) co-receptor. The ternary complex then binds to myeloid differentiated protein 88 (MyD88) to activate nuclear transcription factor kappa B (NF-kB) and mitogen-activated protein kinase (MAPK) signaling pathways and regulate downstream transcription of target genes and generate inflammatory responses. This pathway may be antagonized by IL-36Ra or IL-38. Alternatively, protease inhibition by SERPINA1 or SERPINA3 can prevent the generation of IL-36 agonists. From: Pathophysiology of generalized pustular psoriasis, Young KZ, Sarkar MK, Gudjonsson JE. Experimental Dermatology. 2023, Feb 13. doi: 10.1111/exd.14768. ^©^ 2023 John Wiley & Sons A/S. Reproduced with permission of John Wiley & Sons Ltd.

IL-1 cytokine members are grouped into three subfamilies (IL-1, IL-18, and IL-36) based on their consensus sequence and cognate (i.e. corresponding) receptors; IL-1 and IL-36 subfamilies share accessory protein IL-1R3 (IL-1RAcP) (26) as their co-receptor, while the IL-18 subfamily utilizes a different co-receptor (25). Binding of an agonist to its receptor causes recruitment of the co-receptor, and activation of intracellular signaling pathways that result in increased gene expression of pro-inflammatory mediators (25), as shown for IL-36 in **Figure 2** (29). Formation of the receptor complex recruits intracellular adaptor proteins, including myeloid differentiation primary response 88 (Myd88), IL-1 receptor-associated kinase (IRAK), and TNF receptor-associated factor, which subsequently activate mitogen-activated protein kinase (MAPK) and nuclear factor-κB (NFκB) pathways. NFκB upregulates a broad range of pro-inflammatory gene products. IL-1 and IL-36 cytokines are negatively regulated by their receptor antagonist (IL-1Ra and IL-36Ra, respectively), *via* competitive binding for the receptor site (25). IL-36R is also inhibited by cytokine IL-38, which shares 40% sequence homology with IL-36Ra (and IL-1Ra) (25).

All IL-1 family cytokines are expressed within the skin to some extent (27). IL-36 cytokines are expressed mainly in epithelial and immune cells at barrier sites (skin, lung, and intestine) (24, 30). Receptor agonists IL-36α and IL-36β are present in healthy skin. IL-36γ is constitutively expressed at low levels by keratinocytes, and is upregulated following activation in both PV and GPP lesions (14, 31). IL-36 cytokines are released as precursors, and their activation is carried out by neutrophil-derived proteases (cathepsin G, protease 3, and elastase) (32) and cathepsin S (33).

IL-1 and IL-36 cytokines have similar actions in that their agonists bind to the cognate receptor to trigger pro-inflammatory activity and are inhibited by their respective receptor antagonist. Unopposed IL-1 or IL-36 signaling, which may be caused by loss-of-function mutations in the gene encoding the interleukin receptor antagonist (*IL1RN* or *IL36RN*, respectively), leads to autoinflammatory disease such as Deficiency of the IL-1 Receptor Antagonist (DIRA) (34, 35), and Deficiency of the IL-36 Receptor Antagonist (DITRA) (36, 37). DIRA presents at birth, or soon thereafter, with acute onset pustular dermatitis, systemic inflammation, nail dystrophy, and inflammation of the bone (sterile osteomyelitis) and periosteum; DIRA is rapidly fatal if untreated (34, 35, 38–40). DITRA usually presents in childhood with repeated and severe manifestations of GPP, including high-grade fever, but inflammation of the bone is lacking (36, 37, 40–42). DIRA and DITRA represent autosomal recessive loss-of-function mutations in genes *IL1RN* and *IL36RN*, respectively (**Table 2**) (34–42).

**Table 2:** Genetic and Clinical Features of DIRA and DITRA.

Details	DIRA (Deficiency of IL-1 Receptor Antagonist)	DITRA (Deficiency of IL-36 Receptor Antagonist)
***Overview***	• Autosomal recessive autoinflammatory disease, first reported in 2009 (34, 35) • Loss-of-function mutations in *IL1RN* gene result in lack of functional IL-1 receptor antagonist & unopposed activity of agonists IL-1α/β	• Autosomal recessive autoinflammatory disease, first reported in 2011 (36, 37) • Loss-of-function mutations in *IL36RN* gene result in lack of functional IL-36 receptor antagonist & unopposed activity of agonists IL-36α/β/γ
***Genetics***	• Homozygous or compound heterozygous mutations in *IL1RN* gene producing truncated inactive protein product • Deletion of chromosome segment encompassing *IL1RN* gene • Heterozygous carriers are asymptomatic (34)	• Homozygous, heterozygous, or compound heterozygous mutations in *IL36RN* gene • Predominantly missense substitutions, but also nonsense and frameshift mutations (42) • Homozygous and heterozygous variants in *IL36RN* gene were identified in healthy cohorts
***Clinical presentation***	• Presents at birth or within weeks thereafter • Acute onset of pustular dermatitis, inflammation of the bone and periosteum, systemic inflammation • Fever is usually absent • Rapidly life-threatening if untreated; 30% mortality rate in original case series (34)	• Commonly presents during childhood • Severe manifestation of GPP; repeated episodes of generalized rash and disseminated pustules, systemic inflammation • High-grade fever • Potentially life-threatening complications if untreated
***Effects in mouse model***	• BALB/c mice lacking IL-1 receptor antagonist developed spontaneous skin inflammation, but without full DIRA phenotype (39)	• Transgenic mice expressing *IL1F6* (homologous to *IL-36α* gene) with a deficiency in *IL1F5* (*IL36RN* gene) developed a pustular inflammatory skin disorder (41)
***Treatment***	• Rapid remission in response to anakinra (recombinant IL-1 receptor antagonist), as reported in case series & case reports (40)	• Varying responses to anakinra; other agents have been used (other anti-IL-1, anti-TNF-α, anti-IL-12/23), as reported in case series & case reports (40)

### Genetic background of GPP

**Figure 3:**
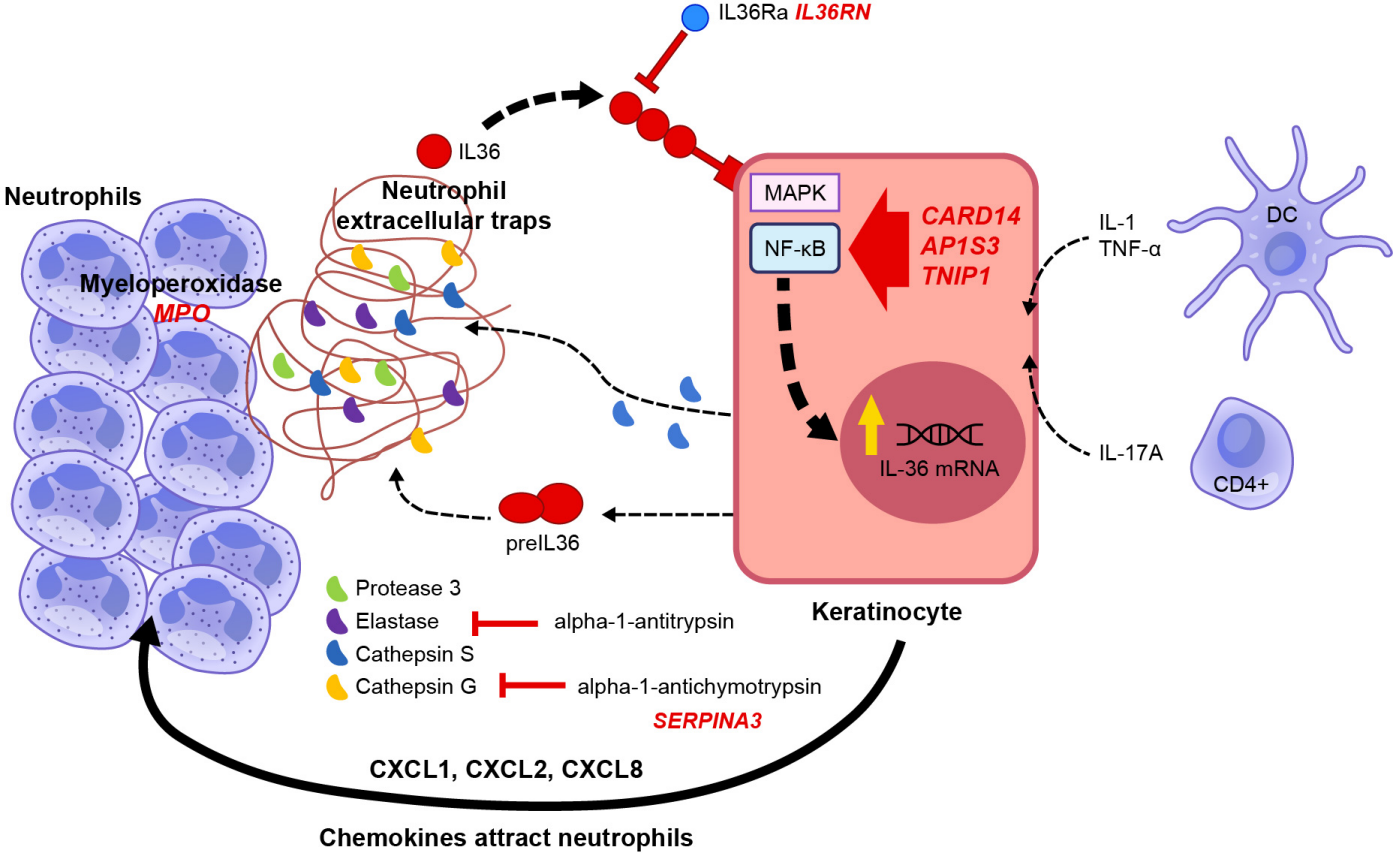
Genetic factors related to GPP pathogenesis (58). Schematic representation of the signal transduction pathway activated by cytokines and genes involved in IL-36 autocrine and autoinflammatory circuits. The pathogenesis of GPP is related to mutations in multiple genes, such as the human IL-1Ra gene (*IL1RN*), IL-36Ra (*IL36RN*), caspase recruitment domain-containing protein 14 (*CARD14*), adapter protein complex 1 subunit sigma 3 (*AP1S3*), TNFAIP3-interacting protein 1 (*TNIP1*), and the gene coding for alpha-1 antichymotrypsin, also known as serine protease inhibitor gene serpin family A member 3 (*SERPINA3*). IL-1, TNF, and IL-17A promote the expression of IL-36 by keratinocytes. IL-36 cytokines are released as precursors requiring enzymatical cleavage by neutrophil-derived proteases (elastase, cathepsin G, or protease 3) and keratinocyte-derived cathepsin S. Mature IL-36 cytokines have 500-fold greater biological activity than their precursors and bind to IL-36R on the keratinocyte cell surface, acting in an autocrine manner to further induce IL-36 expression. In addition, they induce the production and secretion of neutrophil chemokines CXCL1, CXCL2, CXCL6, and CXCL8 (IL-8), increasing the attraction of neutrophils to the skin. Serine protease inhibitors such as alpha-1 antitrypsin or alpha-1 antichymotrypsin (encoded by *SERPINA1* and *SERPINA3*, respectively) can inhibit neutrophil proteases. Adapted from Iznardo et al. (25). From: Generalized Pustular Psoriasis: A Review on Clinical Characteristics, Diagnosis, and Treatment; Rivera-Díaz R, Daudén E, Carrascosa JM, Cueva P, Puig L; Dermatology and Therapy (Heidelb). 2023 Mar;13(3):673-688.

In 2011, homozygous missense mutations in the *IL36RN* gene were identified in nine Tunisian families in which multiple members had GPP (36). Homozygous and compound heterozygous missense *IL36RN* mutations were also identified in three of five unrelated individuals with GPP (without associated PV) (37). Since that discovery, multiple types of mutation have been identified in *IL36RN* and associated with GPP (42–44). *IL36RN* mutations were found far more frequently in patients with GPP alone than in those with GPP plus PV (15, 45–48), and the presence of *IL36RN* mutations was associated with early onset of disease (15, 44, 47). Several other genes have been identified with loss-of-function mutations that are associated with a predisposition to develop GPP; namely, *CARD14* (caspase-activating recruitment domain member 14, also known as *CARMA2*) (49, 50), *AP1S3* (adaptor protein 1 complex subunit sigma 3 (51, 52), *MPO* (myeloperoxidase) (53, 54), *SERPINA3* (serine protease inhibitor A3) (55), and possibly *SERPINA1* (56). These genes are all involved in regulating the IL-1/IL-36-chemokine-neutrophil axis (42, 57), as shown in **Figure 3** (58). However, one study reported that 64% (39/61) of patients with GPP lacked any causal or disease-contributing mutations in *IL36RN, CARD14*, or *AP1S3* genes (59). Also, none of 11 patients with a heterozygous *IL36RN* mutation carried a second non-coding *IL36RN* mutation, suggesting the presence of additional disease-causing genetic factors outside of *IL36RN* alone, but only 15% (3/20) of patients with an *IL36RN* gene mutation also carried a mutation in *CARD14* or *AP1S3* genes (59). Furthermore, no differences in gene expression in the profiles of patients with and without *IL36RN* mutations have been described to date (60). Thus, the genetic basis for GPP is not completely understood.

### IL-36 and GPP in mouse models of skin inflammation

The overexpression of IL-36 in GPP and PV lesional skin in patients is similar to data obtained from mouse models of skin inflammation. Transgenic mice over-expressing *IL1F6* (homologous to *IL36α* gene) and also bearing a deficiency in *IL1F5* (*IL36RN* gene) developed a pustular inflammatory skin phenotype (41). Imiquimod (IMQ), an activator of Toll-like receptor-7, also induces psoriasis-like dermatitis in mice that is mediated *via* the pro-inflammatory IL-23/IL-17 axis (61). IL-36 receptor-deficient mice (*Il36r^–/–^*) were protected from IMQ-induced psoriasis-like dermatitis (62), whereas loss of IL-36Ra (*Il36rn^–/–^*) exacerbated disease severity (63). Additionally, mice deficient in IL-23, IL-17, or IL-22 were not as well protected from disease compared with *Il36r^–/–^* mice, indicating an additional distinct activity of IL-36 beyond induction of the IL-23/IL-17 signaling axis (63). Targeted deletion of *Il36r^–/–^* in mouse keratinocytes resulted in similar protection from IMQ-induced psoriasiform inflammation to that observed in mice with a global deficiency of *Il36r^–/–^*, demonstrating the key role of keratinocytes in IL-36-mediated effects (64). Goldstein et al. showed that *Il36r* signaling in keratinocytes is critical for IL-23 production and controls the recruitment of neutrophils at early treatment time points in the IMQ model (62). These data also indicate that IL-36-dervied signaling in keratinocytes may have an upstream role in the amplified “feed-forward” response by direct regulation of IL-17A expression, potentially *via* IL-23 induction (64). *Il36a* was required for the development of IMQ-induced murine psoriasis, whereas deletion of *Il36a* resulted in significant improvement in the skin lesions (65, 66); however, deficiency of *Il36b* or *Il36g* had no impact on reducing disease severity (66). Also, IL-36α expression was induced by IL-1α, and then acted *via* a feedback loop to induce IL-1α; thus, the two cytokines appear to cooperate to promote psoriasis-like dermatitis in mice (66). Additional investigation of the formation of neutrophil extracellular traps in the IMQ-mouse model demonstrated that neutrophils amplified the epidermal inflammatory responses *via* activation of TLR4/IL-36 cross-talk (67).

However, these findings in the IMQ-mouse model, and other murine models of psoriasis, have significant limitations due to inconsistent laboratory protocols, contradictory findings, and the inability to fully recapitulate complex, multigenic diseases such as psoriasis (68). This underscores the importance of translational human studies, and carefully designed clinical trials that allow for the collection and laboratory evaluation of tissues derived from patients with GPP. Interestingly, mice deficient in *Il36rn* do not exhibit any phenotypic manifestations on their skin, unlike humans with GPP. The only exception is when they are crossed with transgenic mice over-expressing *Il36a* or have IMQ applied to the skin. One hypothesis for this is that IL-8 is a critical cytokine for stimulating neutrophil chemotaxis in skin (only IL-8 activates both CXCR1 and CXCR2) (69). IL-8 is strongly up-regulated by IL-36 in human keratinocytes; thus, strong activation of IL-8 by IL-36 (even in the presence of an active IL-36Ra molecule in humans) may be a key driver of pustulosis. However, cases of *IL36RN* deficiency have even higher activation of IL-8 and the associated neutrophilic axis. Mice do not have a gene for IL-8 and depend on orthologs of IL-8 such as CXCL-1 (which is also present in GPP); thus, mice may require even higher activation of CXCL1 (as IL-8 is absent) to stimulate a neutrophil response in the skin. This threshold may require not only over-expression of IL-36, but also the absence of *IL-36RN*. Furthermore, mice are relatively neutropenic compared to humans; typically, only 5-10% of circulating leukocytes in mice are neutrophils, whereas >50% is more usual in humans (70). This may also suggest a higher threshold for activating a neutrophil-predominant infiltrate in mouse skin. Other murine factors may also contribute to differences in the manifestation of inflammatory skin diseases in mice, such as their predominance of gamma-delta T-lymphocytes, intrinsic differences in the immune system of various genetic strains of mice, method of disease induction (knockout models versus intralesional or topical induction), increased keratinocyte turnover, and thinner skin tissue layers (68).

### The role of IL-36 in skin pathology in GPP and psoriatic disease

**Figure 4:**
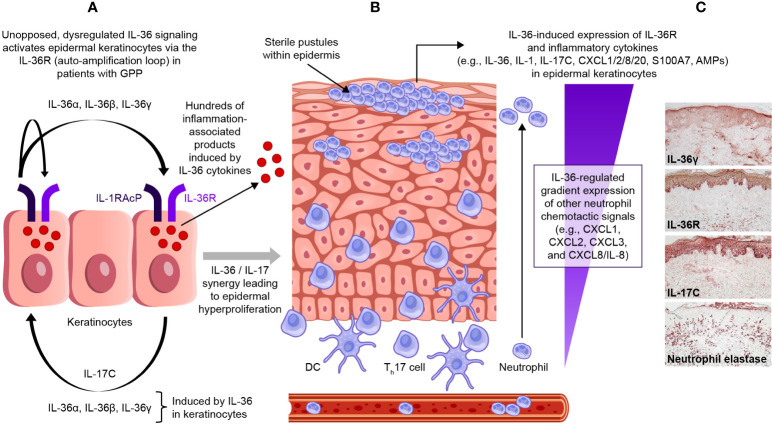
Dysregulated IL-36 signaling in epidermal keratinocytes in GPP. In patients with GPP, unopposed, dysregulated IL-36 signaling activates epidermal keratinocytes *via* the IL-36R (auto-amplification loop) **(A)**. This induces the release of a wide range of inflammation-associated products, and leads to a gradient of IL-36-regulated neutrophil chemotactic signals **(B)**. The expression of IL-36R is strong and dominant on epidermal keratinocytes within the viable epidermal layers **(C)**. GPP, generalized pustular psoriasis; IL-36R, interleukin-36 receptor. [Panel **C**, immunohistochemical sample was kindly provided by Dr JG Krueger.].

IL-36 cytokines (IL-36α, IL-36β, and IL-36γ) maintain circuits that recruit and activate neutrophils, and this process plays a central role in pathogenesis of GPP (71), as shown in **Figure 4**. Under normal conditions, IL-36 agonist activity is balanced by a high level of IL-36Ra, while IL-36R remains inactive or signals at a low-level, and this equilibrium maintains a regulated downstream inflammatory response (72). However, if the IL-36 axis becomes hyperactivated (due to increased IL-36 agonist, or impaired IL-36Ra activity from *IL36RN* mutation), uncontrolled pro-inflammatory responses ensue, amplifying neutrophil chemotaxis and neutrophil-driven inflammatory responses in the skin (18). The detection of IL-36α and IL-36γ overexpression in lesional skin and peripheral blood samples from patients with GPP further supports the central role of IL-36 dysregulation in this condition (14, 73), and there is a strong association between loss-of-function mutations in *IL36RN* (producing faulty IL-36Ra protein) and increased susceptibility to GPP (36, 37, 46).

It is likely that the IL-36 pathway plays a major role in regulating inflammatory responses at barrier epithelia, such as keratinocytes in the epidermis. From immunohistochemical (IHC) staining of human skin samples, IL-36R expression is strong and dominant on epidermal keratinocytes within the viable epidermal layers, and this pan-epidermal expression is maintained in both PV and in GPP lesions (74). In contrast, IHC staining of dermal cell types, such as fibroblasts and vascular cells, shows IL-36R expression is relatively weak in human skin (74). From *in vitro* experiments with cultured keratinocytes (representing a model where the agonist actions of IL-36 cytokines are relatively unopposed, due to the relative absence of IL-36Ra in the model), it is clear that IL-36 can self-induce expression of all IL-36 ligands (IL-36α, IL-36β, and IL-36γ), but also expression of other inflammatory cytokines such as TNF, IL-6, IL-8, S100A7 (psoriasin), defensins, and other anti-microbial proteins (AMPs) (75). There is also a strong synergistic interaction between IL-36 ligands and IL-17 isoforms, such as IL-17A, that are produced by activated T-cells for these gene products. The role of IL-17A in GPP disease is supported in part by case reports and small clinical trials from Japan detailing the clinical improvement and resolution of systemic symptoms in some patients with GPP treated with selective IL-17A (secukinumab and ixekizumab) and IL-17R (brodalumab) antagonists (76–80).

The breadth of the keratinocyte response to IL-36 family cytokines was only appreciated recently by whole-genome gene profiling experiments using IL-36γ and IL-17A cytokines in human keratinocytes (74). These experiments show that both IL-17A and IL-36γ induce hundreds of gene products that are only partly overlapping, while the response to these combined cytokines is up-regulation of several thousand gene products that are also highly characteristic of genes activated in PV and GPP. Included in this response are numerous chemokines that regulate neutrophil chemotaxis in skin (CXCL1, CXCL2, CXCL3, and CXCL8/IL-8), attract T-cells or inflammatory dendritic cells into skin (CXCL9 and CCL20), and induce or further amplify IL-36 synthesis/signaling in the skin (IL-17C). In addition, IL-1, IL-6, IL-19, and IL-24 are also induced, which can promote broad inflammatory responses as well as induce keratinocyte proliferation (74). Thus, the response of keratinocytes to IL-36 agonists includes increased expression of broad AMPs, the production of inflammatory cytokines and chemokines, and the broad-modification of keratinocyte gene expression programs to mimic psoriatic tissues. **Figure 4C** shows increased staining of IL-36γ, IL-36R, and IL-17C in the epidermis of GPP lesions, along with the influx of neutrophils into the dermis and epidermis of lesions (neutrophil elastase stain). There are extensive overlaps between gene sets induced in cultured keratinocytes by IL-36 and gene expression in GPP, but especially for CXCL chemokines that are induced by IL-36 that would be expected to regulate neutrophil trafficking (74). In addition, abnormal IL-36 activity was found to correlate with a prominent IFN-1 signature in patients with GPP and PV, and IFN-1 gene activation was also associated with extracutaneous morbidity (acute systemic flares in GPP and chronic systemic inflammation in PV) (81). However, despite the significant overlapping immune functions of IL-36 and IL-17 cytokines in the skin, the predominant role of IL-36 signaling in GPP is underscored by the variable and inconsistent clinical efficacy of off-label IL-17 antagonists for the treatment of GPP; in contrast, most patients with PV have consistent clinical responses to selective IL-17 and IL-23 inhibitors. While IL-36 could have a pathogenic role in PV, clinical studies investigating the clinical efficacy of IL-36 blockade in PV patients are lacking and are necessary to determine the exact contribution of IL-36 signaling in plaque and non-GPP subtypes.

Studies of the effects of IL-36 on inflammatory gene expression in peripheral blood mononuclear cells (PBMCs) showed that IL-36 induced expression of TNF, IL-1, IL-6, and IL-8 in normal control PBMCs, but that much higher induction of these cytokines occurred in PBMCs from a patient with a mutation in *IL36RN* (37). Thus, effects of amplified IL-36 signaling in GPP patients likely extends to many connective tissue and blood leukocyte cell types in which expression of IL-36R has been identified. Patients with GPP have high levels of these inflammatory cytokines in their blood prior to treatment (causing high fever, leukocytosis, and systemic inflammatory symptoms), and this may be driven by the extended release of IL-36-induced cytokines produced by various cell types under intensified IL-36 signaling in GPP.

## Targeted inhibition of the IL-36 receptor for the treatment of GPP

Selective blockade of the IL-36 pathway *via* targeted inhibition of IL-36R is an attractive therapeutic strategy for the treatment of GPP, and other diseases involving dysregulated IL-36 signaling. Spesolimab (SPEVIGO^®^; Boehringer Ingelheim Pharmaceuticals, Inc., Ridgefield, CT, USA) is a first-in-class humanized monoclonal immunoglobulin G1 antibody that binds specifically to IL-36R and antagonizes IL-36 signaling (82). Spesolimab was approved by the US FDA in September 2022 to treat GPP flares in adults (21, 22), and regulatory approval in numerous other countries has followed (83). Key clinical trials data that supported FDA approval of spesolimab were the phase 1 proof-of-concept trial (NCT02978690; N = 7) and the phase 2 randomized, placebo-controlled trial, Effisayil™ 1 (NCT03782792; N = 53), the results of which have been published in detail (84–87). In Effisayil™ 1, spesolimab was administered as a single 900 mg dose *via* intravenous (IV) infusion over 90 minutes, with the option of a second 900 mg dose IV given 1 week later if symptoms persist (85). Spesolimab efficacy was assessed *via* the GPP Physician Global Assessment (GPPGA), and the GPP Area and Severity Index (GPPASI) (88). Biomarkers in skin and blood were evaluated (60, 89), and patient-reported outcome instruments were reported (90). In the proof-of-concept and Effisayil™ 1 trials, spesolimab treatment led to rapid (within one week) and sustained (to end of trial) clinical improvement and pustular clearance in patients with GPP flares, and was safe and well-tolerated (84, 85). Efficacy and safety were also consistent for the trial duration across prespecified subgroups in Effisayil™ 1 (91, 92). Pyrexia was observed in both spesolimab (6%) and placebo (22%) groups during Effisayil^™^ 1 (85), suggesting that this reported adverse event was more likely GPP-associated rather than a treatment-specific effect. Infections were the most frequent adverse reactions observed in patients treated with spesolimab (22); during the 1-week placebo-controlled period in Effisayil™ 1, infections were reported in 14% of the spesolimab group versus 6% of the placebo group (22). Effisayil™ 2 and Effisayil^®^ ON are additional clinical trials to investigate the efficacy and safety of spesolimab in patients with a history of GPP. Effisayil™ 2 (NCT04399837; N = 123 [ (93)]) was completed in December 2022, and was published recently (94). Effisayil™ ON (NCT03886246; N = 131 [ (95)]) is an active 5-year open-label extension study, in which participants of Effisayil™ 1 and Effisayil™ 2 were recruited (96).

### Gene expression profiles following spesolimab treatment in GPP

**Figure 5:**
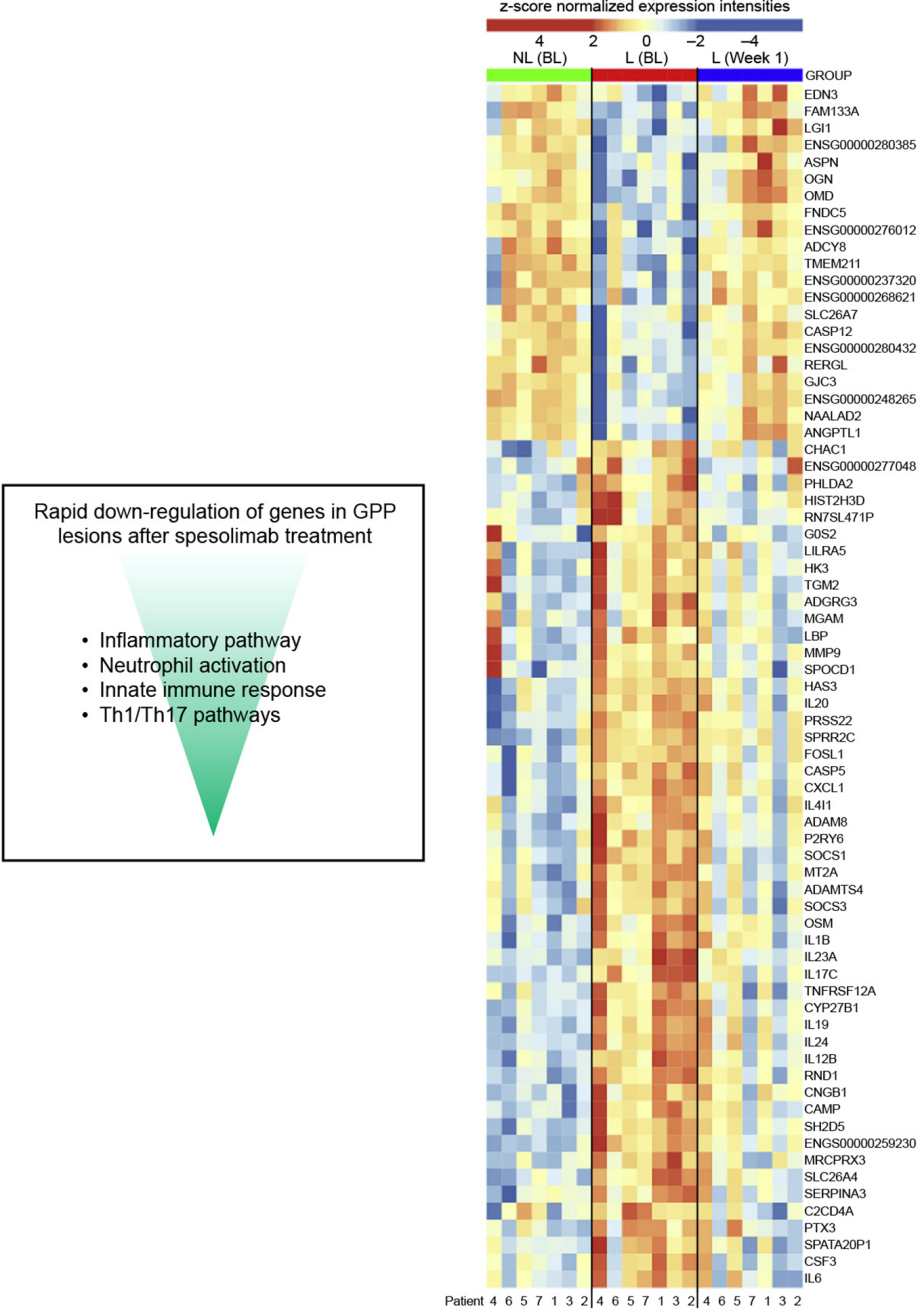
The effect of spesolimab on differential gene expression in GPP (60). Heatmap of 71 differentially expressed genes that are deregulated between lesional (L) and nonlesional (NL) skin at baseline (BL), and lesional skin 1 week after treatment with spesolimab compared with lesional skin at baseline (absolute log_2_ fold change ≥ 2, adjusted P ≤.05). GPP, generalized pustular psoriasis. From: Pustular psoriasis: Molecular pathways and effects of spesolimab in generalized pustular psoriasis; Baum P, Visvanathan S, Garcet S, Roy J, Schmid R, Bossert S, et al; J Allergy Clin Immunol. 2022;149(4):1402-12.

Pre- and post-treatment skin and blood samples were collected from participants in the phase 1 proof-of-concept trial and Effisayil^®^ 1 trial to compare gene expression profiles in GPP lesions versus non-lesional skin, and assess molecular changes before versus after spesolimab treatment (60, 97). In lesional skin, spesolimab treatment led to significant decreases in the expression of genes associated with pro-inflammatory mediators (e.g. *TNF*, *IL1B*, *IL6*), neutrophil recruitment (e.g. *CXCR1*, *CXCR2*), keratinocyte-mediated inflammation and proliferation (e.g. IL20), and IL-36 ligands (*IL36A*, *IL36B*, *IL36G*) (60, 97), as shown in **Figure 5** (60). Reductions in select serum biomarker levels were also identified; including those linked to inflammation (C-reactive protein, TNFα), neutrophilic markers (CXCL1, IL-8), innate pathways (IL-1RN, IL-6), and Th17 pathways (IL-17A, CCL20) (89). These reductions were associated with clinical improvement in GPP, as assessed by the respective primary endpoints of each trial (89). These changes in gene expression and protein biomarker data demonstrate that spesolimab treatment reverses the lesional skin molecular profile associated with GPP.

### Other investigational agents in the treatment of GPP

A second IL-36 receptor blocker, imsidolimab [AnaptysBio, Inc., San Diego, CA, USA (98)], completed a phase 2 clinical trial in patients with active moderate-to-severe GPP (GALLOP; NCT03619902; N = 8) (99, 100). Participants received imsidolimab 750 mg *via* IV infusion, followed by three further doses of 100 mg given subcutaneously at approximately 30-day intervals, with a 12-week follow-up. Six of the eight participants achieved the primary endpoint of improvement in Clinical Global Impression (based on the modified Japanese Dermatology Association Severity Index) at Week 4 and at Week 16 (100). A phase 3 clinical trial of imsidolimab in patients with GPP flare is now being conducted (GEMINI-1; NCT05352893; N = 45), with a long-term extension trial to follow (GEMINI-2; NCT05366855; N = 45 planned), and top-line data from GEMINI-1 are expected by the end of 2023 (101). Other investigational agents that may target the IL-36 pathway include REGN6490 (Regeneron), an antibody that blocks the IL-36 receptor, and the small molecule A-522 (AbbVie, Inc.) that antagonizes IL-36γ (102). However, phase 1 development of REGN6490 was terminated recently (103, 104).

## Unmet needs in GPP and the future of IL-36 pathway modulation

Several other pustular skin conditions can mimic the clinical presentation of GPP (105); such as acute generalized exanthematous pustulosis (AGEP), other severe drug reactions, infections, IgA pemphigus, subcorneal pustular dermatosis (also called Sneddon-Wilkinson disease), acute psoriasis subtypes (e.g. erythrodermic and pustular disease flares), and localized forms of pustular psoriasis, such as palmoplantar pustulosis (PPP). Though IL-36 cytokines are highly expressed in AGEP lesional skin, the precise relationship between AGEP and IL-36 signaling is unclear (106–108). *IL36RN* mutations have been detected in a small proportion of patients with PPP, but the relationship is unclear given the lack of response in patients given an IL-36R inhibitor (15, 109, 110).

Consequently, GPP is often misdiagnosed, or may go unrecognized by clinicians who are unfamiliar with managing this disease. Patients often present in the emergency room or urgent care clinics with severe manifestations of GPP (111), which, if not treated effectively, may result in complications such as infection/sepsis, renal and liver dysfunction, or even death (112). Current biologic treatments approved for PV are often ineffective at controlling GPP; thus, GPP-specific treatment is needed to prevent and control flares. Furthermore, clinicians may be unable or unwilling to prescribe biologics that are not specifically FDA-approved for the treatment of GPP (111), and these agents are inconsistently effective for the treatment of GPP. In the CorEvitas (formerly Corrona) Psoriasis Patient Registry study, 67% of dermatologists (N = 29) reported that flare prevention was a challenge when treating patients with frequent flares (two or more episodes per year) (111). Thus, multi-level support from dermatologists, emergency physicians, and primary care providers is needed to facilitate rapid decision-making during acute illness to control skin and systemic symptoms in patients with GPP (113). Consensus guidelines for the diagnosis and management of GPP are also lacking, but are greatly needed to broaden disease understanding and improve disease management by healthcare professionals (111, 113). Importantly, the recent development of an evidence-based clinical management algorithm is now available, following a recent Delphi panel involving 21 expert dermatologists who established global consensus on the clinical course, diagnosis, treatment goals, and disease management of GPP (114).

In addition to the central role of IL-36 receptor signaling in the pathogenesis of GPP, evidence suggests that IL-36 may have a role in the pathogenesis of other diseases (115, 116); including inflammatory bowel disease (117, 118), hidradenitis suppurativa (119, 120), arthritis (121–123), systemic lupus erythematosus (124, 125), pyoderma gangrenosum (126), and Netherton syndrome (127). Phase 2 clinical trials of spesolimab in the treatment of hidradenitis suppurativa are active (NCT04876391), or recently completed (NCT04762277 (128), and a Phase 2/3 clinical trial of spesolimab in the treatment of Netherton syndrome is in progress (NCT05856526). However, a recent Phase 2b randomized placebo-controlled trial of spesolimab in patients with PPP did not meet its primary endpoint (110). Investigations are also underway into the possible role of IL-36 cytokines in fibrotic disorders (115) and malignancy (129–131).

## Conclusions

Dysfunctional elements in the IL-36 pathway drive the clinical features and symptoms of GPP. Targeted inhibition of IL-36R (via spesolimab, imsidolimab, or other novel agents) is an attractive therapeutic strategy for the treatment of GPP. Therapies that target IL-36R, such as spesolimab, could mitigate flares, address flare prevention, and provide sustained disease control in patients with GPP, presumably through rebalancing IL-36 signaling and modulating the pro-inflammatory response of downstream effectors. However, investigation is needed to explain why patients with the same *IL36RN* mutations can present with differences in flare severity and frequency, and to better understand the underlying differences in the disease mechanisms in patients with GPP who do not have identifiable causal or disease-contributing mutations.

## Author contributions

JH: Writing – original draft, Writing – review & editing. SV: Writing – original draft, Writing – review & editing. JK: Writing – original draft, Writing – review & editing.

## References

[1] Navarini AA Burden AD Capon F Mrowietz U Puig L Koks S. European consensus statement on phenotypes of pustular psoriasis. J Eur Acad Dermatol Venereol (2017) 31(11):1792–9. doi: 10.1111/jdv.14386 28585342

[2] Kharawala S Golembesky AK Bohn RL Esser D. The clinical, humanistic, and economic burden of generalized pustular psoriasis: a structured review. Expert Rev Clin Immunol (2020) 16(3):239–52. doi: 10.1080/1744666X.2019.1708193 32073341

[3] Choon SE Navarini AA Pinter A. Clinical course and characteristics of generalized pustular psoriasis. Am J Clin Dermatol (2022) 23(Suppl 1):21–9. doi: 10.1007/s40257-021-00654-z PMC8801409 35061227

[4] Koren J Mburu S Trigos D Damiani G Naldi L. Generalised pustular psoriasis: the case for rare disease and orphan designation. Br J Dermatol (2022) 187(3):411–3. doi: 10.1111/bjd.21231 PMC9542987 35257372

[5] Augey F Renaudier P Nicolas JF. Generalized pustular psoriasis (Zumbusch): a French epidemiological survey. Eur J Dermatol (2006) 16(6):669–73. 17229609

[6] Ohkawara A Yasuda H Kobayashi H Inaba Y Ogawa H Hashimoto I. Generalized pustular psoriasis in Japan: two distinct groups formed by differences in symptoms and genetic background. Acta Derm Venereol (1996) 76(1):68–71. doi: 10.2340/00015555766871 8721499

[7] Lee JY Kang S Park JS Jo SJ. Prevalence of psoriasis in korea: A population-based epidemiological study using the korean national health insurance database. Ann Dermatol (2017) 29(6):761–7. doi: 10.5021/ad.2017.29.6.761 PMC5705359 29200766

[8] Prinz JC Choon SE Griffiths CEM Merola JF Morita A Ashcroft DM. Prevalence, comorbidities and mortality of generalized pustular psoriasis: A literature review. J Eur Acad Dermatol Venereol (2023) 37(2):256–73. doi: 10.1111/jdv.18720 36331364

[9] Choon SE Lai NM Mohammad NA Nanu NM Tey KE Chew SF. Clinical profile, morbidity, and outcome of adult-onset generalized pustular psoriasis: analysis of 102 cases seen in a tertiary hospital in Johor, Malaysia. Int J Dermatol (2014) 53(6):676–84. doi: 10.1111/ijd.12070 23967807

[10] Miyachi H Konishi T Kumazawa R Matsui H Shimizu S Fushimi K. Treatments and outcomes of generalized pustular psoriasis: A cohort of 1516 patients in a nationwide inpatient database in Japan. J Am Acad Dermatol (2022) 86(6):1266–74. doi: 10.1016/j.jaad.2021.06.008 34116101

[11] Brenner M Molin S Ruebsam K Weisenseel P Ruzicka T Prinz JC. Generalized pustular psoriasis induced by systemic glucocorticosteroids: four cases and recommendations for treatment. Br J Dermatol (2009) 161(4):964–6. doi: 10.1111/j.1365-2133.2009.09348.x 19681858

[12] Benzaquen M Flachaire B Rouby F Berbis P Guis S. Paradoxical pustular psoriasis induced by ustekinumab in a patient with Crohn’s disease-associated spondyloarthropathy. Rheumatol Int (2018) 38(7):1297–9. doi: 10.1007/s00296-018-4034-0 29705819

[13] Balak DM Hajdarbegovic E. Drug-induced psoriasis: clinical perspectives. Psoriasis (Auckl) (2017) 7:87–94. doi: 10.2147/ptt.S126727 29387611 PMC5774610

[14] Johnston A Xing X Wolterink L Barnes DH Yin Z Reingold L. IL-1 and IL-36 are dominant cytokines in generalized pustular psoriasis. J Allergy Clin Immunol (2017) 140(1):109–20. doi: 10.1016/j.jaci.2016.08.056 PMC5494022 28043870

[15] Twelves S Mostafa A Dand N Burri E Farkas K Wilson R. Clinical and genetic differences between pustular psoriasis subtypes. J Allergy Clin Immunol Mar (2019) 143(3):1021–6. doi: 10.1016/j.jaci.2018.06.038 PMC6403101 30036598

[16] Bachelez H Barker J Burden AD Navarini AA Krueger JG. Generalized pustular psoriasis is a disease distinct from psoriasis vulgaris: evidence and expert opinion. Expert Rev Clin Immunol (2022) 18(10):1033–47. doi: 10.1080/1744666X.2022.2116003 36062811

[17] Borges-Costa J Silva R Goncalves L Filipe P Soares de Almeida L Marques Gomes M. Clinical and laboratory features in acute generalized pustular psoriasis: a retrospective study of 34 patients. Am J Clin Dermatol (2011) 12(4):271–6. doi: 10.2165/11586900-000000000-00000 21495733

[18] Uppala R Tsoi LC Harms PW Wang B Billi AC Maverakis E. “Autoinflammatory psoriasis”-genetics and biology of pustular psoriasis. Cell Mol Immunol (2021) 18(2):307–17. doi: 10.1038/s41423-020-0519-3 PMC8027616 32814870

[19] Hawkes JE Chan TC Krueger JG. Psoriasis pathogenesis and the development of novel targeted immune therapies. J Allergy Clin Immunol (2017) 140(3):645–53. doi: 10.1016/j.jaci.2017.07.004 PMC5600287 28887948

[20] Grän F Kerstan A Serfling E Goebeler M Muhammad K. Current developments in the immunology of psoriasis. Yale J Biol Med (2020) 93(1):97–110. 32226340 PMC7087066

[21] Boehringer Ingelheim. FDA approves the first treatment option for generalized pustular psoriasis flares in adults (2022). Available at: https://www.boehringer-ingelheim.us/press-release/fda-approves-first-treatment-option-generalized-pustular-psoriasis-flares-adults (Accessed September 28, 2022).

[22] Boehringer Ingelheim. SPEVIGO prescribing information (2022). Available at: https://www.accessdata.fda.gov/drugsatfda_docs/label/2022/761244s000lbl.pdf (Accessed September 28, 2022).

[23] Garlanda C Dinarello CA Mantovani A. The interleukin-1 family: back to the future. Immunity (2013) 39(6):1003–18. doi: 10.1016/j.immuni.2013.11.010 PMC3933951 24332029

[24] Iznardo H Puig L. Exploring the role of IL-36 cytokines as a new target in psoriatic disease. Int J Mol Sci (2021) 22(9):4344. doi: 10.3390/ijms22094344 33919434 PMC8122427

[25] Iznardo H Puig L. The interleukin-1 family cytokines in psoriasis: pathogenetic role and therapeutic perspectives. Expert Rev Clin Immunol (2021) 17(2):187–99. doi: 10.1080/1744666X.2021.1886081 33538202

[26] Dinarello CA. Overview of the IL-1 family in innate inflammation and acquired immunity. Immunol Rev (2018) 281(1):8–27. doi: 10.1111/imr.12621 29247995 PMC5756628

[27] Macleod T Berekmeri A Bridgewood C Stacey M McGonagle D Wittmann M. The immunological impact of IL-1 family cytokines on the epidermal barrier. Front Immunol (2021) 12:808012. doi: 10.3389/fimmu.2021.808012 35003136 PMC8733307

[28] Green EA Garrick SP Peterson B Berger PJ Galinsky R Hunt RW. The role of the interleukin-1 family in complications of prematurity. Int J Mol Sci (2023) 24(3):2795. doi: 10.3390/ijms24032795 36769133 PMC9918069

[29] Young KZ Sarkar MK Gudjonsson JE. Pathophysiology of generalized pustular psoriasis. Exp Dermatol (2023) 32(8):1194–203. doi: 10.1111/exd.14768 PMC10423307 36779688

[30] Zhou L Todorovic V. Interleukin-36: structure, signaling and function. Adv Exp Med Biol (2021) 21:191–210. doi: 10.1007/5584_2020_488 32026417

[31] Macleod T Ainscough JS Hesse C Konzok S Braun A Buhl AL. The proinflammatory cytokine IL-36gamma is a global discriminator of harmless microbes and invasive pathogens within epithelial tissues. Cell Rep (2020) 33(11):108515. doi: 10.1016/j.celrep.2020.108515 33326792 PMC7758160

[32] Henry CM Sullivan GP Clancy DM Afonina IS Kulms D Martin SJ. Neutrophil-derived proteases escalate inflammation through activation of IL-36 family cytokines. Cell Rep (2016) 14(4):708–22. doi: 10.1016/j.celrep.2015.12.072 26776523

[33] Ainscough JS Macleod T McGonagle D Brakefield R Baron JM Alase A. Cathepsin S is the major activator of the psoriasis-associated proinflammatory cytokine IL-36gamma. Proc Natl Acad Sci U S A (2017) 114(13):E2748–57. doi: 10.1073/pnas.1620954114 PMC5380102 28289191

[34] Aksentijevich I Masters SL Ferguson PJ Dancey P Frenkel J van Royen-Kerkhoff A. An autoinflammatory disease with deficiency of the interleukin-1-receptor antagonist. N Engl J Med (2009) 360(23):2426–37. doi: 10.1056/NEJMoa0807865 PMC2876877 19494218

[35] Reddy S Jia S Geoffrey R Lorier R Suchi M Broeckel U. An autoinflammatory disease due to homozygous deletion of the IL1RN locus. N Engl J Med (2009) 360(23):2438–44. doi: 10.1056/NEJMoa0809568 PMC2803085 19494219

[36] Marrakchi S Guigue P Renshaw BR Puel A Pei XY Fraitag S. Interleukin-36-receptor antagonist deficiency and generalized pustular psoriasis. N Engl J Med (2011) 365(7):620–8. doi: 10.1056/NEJMoa1013068 21848462

[37] Onoufriadis A Simpson MA Pink AE Di Meglio P Smith CH Pullabhatla V. Mutations in IL36RN/IL1F5 are associated with the severe episodic inflammatory skin disease known as generalized pustular psoriasis. Am J Hum Genet (2011) 89(3):432–7. doi: 10.1016/j.ajhg.2011.07.022 PMC3169817 21839423

[38] Martin P Goldstein JD Mermoud L Diaz-Barreiro A Palmer G. IL-1 family antagonists in mouse and human skin inflammation. Front Immunol (2021) 12:652846. doi: 10.3389/fimmu.2021.652846 33796114 PMC8009184

[39] Shepherd J Little MC Nicklin MJ. Psoriasis-like cutaneous inflammation in mice lacking interleukin-1 receptor antagonist. J Invest Dermatol (2004) 122(3):665–9. doi: 10.1111/j.0022-202X.2004.22305.x 15086551

[40] Gomez-Garcia F Sanz-Cabanillas JL Viguera-Guerra I Isla-Tejera B Nieto AV Ruano J. Scoping review on use of drugs targeting interleukin 1 pathway in DIRA and DITRA. Dermatol Ther (Heidelb) (2018) 8(4):539–56. doi: 10.1007/s13555-018-0269-7 PMC6261121 30392030

[41] Blumberg H Dinh H Trueblood ES Pretorius J Kugler D Weng N. Opposing activities of two novel members of the IL-1 ligand family regulate skin inflammation. J Exp Med (2007) 204(11):2603–14. doi: 10.1084/jem.20070157 PMC2118475 17908936

[42] Zhou J Luo Q Cheng Y Wen X Liu J. An update on genetic basis of generalized pustular psoriasis (Review). Int J Mol Med (2021) 47(6):118. doi: 10.3892/ijmm.2021.4951 33955502 PMC8083806

[43] Setta-Kaffetzi N Navarini AA Patel VM Pullabhatla V Pink AE Choon SE. Rare pathogenic variants in IL36RN underlie a spectrum of psoriasis-associated pustular phenotypes. J Invest Dermatol (2013) 133(5):1366–9. doi: 10.1038/jid.2012.490 23303454

[44] Hussain S Berki DM Choon SE Burden AD Allen MH Arostegui JI. IL36RN mutations define a severe autoinflammatory phenotype of generalized pustular psoriasis. J Allergy Clin Immunol (2015) 135(4):1067–70.e9. doi: 10.1016/j.jaci.2014.09.043 25458002

[45] Capon F. IL36RN mutations in generalized pustular psoriasis: just the tip of the iceberg? J Invest Dermatol (2013) 133(11):2503–4. doi: 10.1038/jid.2013.361 24129779

[46] Sugiura K Takemoto A Yamaguchi M Takahashi H Shoda Y Mitsuma T. The majority of generalized pustular psoriasis without psoriasis vulgaris is caused by deficiency of interleukin-36 receptor antagonist. J Invest Dermatol (2013) 133(11):2514–21. doi: 10.1038/jid.2013.230 23698098

[47] Liu ZJ Tian YT Shi BY Zhou Y Jia XS. Association between mutation of interleukin 36 receptor antagonist and generalized pustular psoriasis: A PRISMA-compliant systematic review and meta-analysis. Med (Baltimore) (2020) 99(45):e23068. doi: 10.1097/MD.0000000000023068 PMC7647532 33157966

[48] Berki DM Mahil SK Burden AD Trembath RC Smith CH Capon F. Loss of IL36RN function does not confer susceptibility to psoriasis vulgaris. J Invest Dermatol (2014) 134(1):271–3. doi: 10.1038/jid.2013.285 23792462

[49] Berki DM Liu L Choon SE David Burden A Griffiths CEM Navarini AA. Activating CARD14 mutations are associated with generalized pustular psoriasis but rarely account for familial recurrence in psoriasis vulgaris. J Invest Dermatol Dec (2015) 135(12):2964–70. doi: 10.1038/jid.2015.288 26203641

[50] Jordan CT Cao L Roberson ED. Rare and common variants in CARD14, encoding an epidermal regulator of NF-kappaB, in psoriasis. Am J Hum Genet (2012) 90(5):796–808. doi: 10.1016/j.ajhg.2012.03.013 22521419 PMC3376540

[51] Setta-Kaffetzi N Simpson MA Navarini AA Patel VM Lu HC Allen MH. AP1S3 mutations are associated with pustular psoriasis and impaired Toll-like receptor 3 trafficking. Am J Hum Genet (2014) 94(5):790–7. doi: 10.1016/j.ajhg.2014.04.005 PMC4067562 24791904

[52] Mahil SK Twelves S Farkas K Setta-Kaffetzi N Burden AD Gach JE. AP1S3 mutations cause skin autoinflammation by disrupting keratinocyte autophagy and up-regulating IL-36 production. J Invest Dermatol (2016) 136(11):2251–9. doi: 10.1016/j.jid.2016.06.618 PMC5070969 27388993

[53] Vergnano M Mockenhaupt M Benzian-Olsson N Paulmann M Grys K Mahil SK. Loss-of-function myeloperoxidase mutations are associated with increased neutrophil counts and pustular skin disease. Am J Hum Genet (2020) 107(3):539–43. doi: 10.1016/j.ajhg.2020.06.020 PMC7477255 32758448

[54] Haskamp S Bruns H Hahn M Hoffmann M Gregor A Lohr S. Myeloperoxidase modulates inflammation in generalized pustular psoriasis and additional rare pustular skin diseases. Am J Hum Genet (2020) 107(3):527–38. doi: 10.1016/j.ajhg.2020.07.001 PMC7477008 32758447

[55] Frey S Sticht H Wilsmann-Theis D Gerschutz A Wolf K Lohr S. Rare loss-of-function mutation in SERPINA3 in generalized pustular psoriasis. J Invest Dermatol (2020) 140(7):1451–5 e13. doi: 10.1016/j.jid.2019.11.024 31945348

[56] Kantaputra P Chaowattanapanit S Kiratikanon S Chaiwarith R Choonhakarn C Intachai W. SERPINA1, generalized pustular psoriasis, and adult-onset immunodeficiency. J Dermatol (2021) 48(10):1597–601. doi: 10.1111/1346-8138.16081 34390020

[57] Akiyama M. Pustular psoriasis as an autoinflammatory keratinization disease (AiKD): Genetic predisposing factors and promising therapeutic targets. J Dermatol Sci (2022) 105(1):11–7. doi: 10.1016/j.jdermsci.2021.11.009 34973880

[58] Rivera-Diaz R Dauden E Carrascosa JM Cueva P Puig L. Generalized pustular psoriasis: A review on clinical characteristics, diagnosis, and treatment. Dermatol Ther (Heidelb) (2023) 13(3):673–88. doi: 10.1007/s13555-022-00881-0 PMC9836924 36635445

[59] Mössner R Wilsmann-Theis D Oji V Gkogkolou P Lohr S Schulz P. The genetic basis for most patients with pustular skin disease remains elusive. Br J Dermatol (2018) 178(3):740–8. doi: 10.1111/bjd.15867 28887889

[60] Baum P Visvanathan S Garcet S Roy J Schmid R Bossert S. Pustular psoriasis: Molecular pathways and effects of spesolimab in generalized pustular psoriasis. J Allergy Clin Immunol (2022) 149(4):1402–12. doi: 10.1016/j.jaci.2021.09.035 34678325

[61] van der Fits L Mourits S Voerman JS Kant M Boon L Laman JD. Imiquimod-induced psoriasis-like skin inflammation in mice is mediated *via* the IL-23/IL-17 axis. J Immunol (2009) 182(9):5836–45. doi: 10.4049/jimmunol.0802999 19380832

[62] Goldstein JD Bassoy EY Caruso A Palomo J Rodriguez E Lemeille S. IL-36 signaling in keratinocytes controls early IL-23 production in psoriasis-like dermatitis. Life Sci Alliance (2020) 3(6):e202000688. doi: 10.26508/lsa.202000688 32345660 PMC7190273

[63] Tortola L Rosenwald E Abel B Blumberg H Schafer M Coyle AJ. Psoriasiform dermatitis is driven by IL-36-mediated DC-keratinocyte crosstalk. J Clin Invest (2012) 122(11):3965–76. doi: 10.1172/JCI63451 PMC3484446 23064362

[64] Hernandez-Santana YE Leon G St Leger D Fallon PG Walsh PT. Keratinocyte interleukin-36 receptor expression orchestrates psoriasiform inflammation in mice. Life Sci Alliance (2020) 3(4):e201900586. doi: 10.26508/lsa.201900586 32086318 PMC7035875

[65] Campbell JJ Ebsworth K Ertl LS McMahon JP Wang Y Yau S. Efficacy of chemokine receptor inhibition in treating IL-36alpha-induced psoriasiform inflammation. J Immunol (2019) 202(6):1687–92. doi: 10.4049/jimmunol.1801519 30718298

[66] Milora KA Fu H Dubaz O Jensen LE. Unprocessed interleukin-36alpha regulates psoriasis-like skin inflammation in cooperation with interleukin-1. J Invest Dermatol (2015) 135(12):2992–3000. doi: 10.1038/jid.2015.289 26203636 PMC4648684

[67] Shao S Fang H Dang E Xue K Zhang J Li B. Neutrophil extracellular traps promote inflammatory responses in psoriasis *via* activating epidermal TLR4/IL-36R crosstalk. Front Immunol (2019) 10:746. doi: 10.3389/fimmu.2019.00746 31024570 PMC6460719

[68] Hawkes JE Adalsteinsson JA Gudjonsson JE Ward NL. Research techniques made simple: murine models of human psoriasis. J Invest Dermatol (2018) 138(1):e1–8. doi: 10.1016/j.jid.2017.10.013 PMC6902858 29273150

[69] Schraufstatter IU Chung J Burger M. IL-8 activates endothelial cell CXCR1 and CXCR2 through Rho and Rac signaling pathways. Am J Physiol Lung Cell Mol Physiol (2001) 280(6):L1094–103. doi: 10.1152/ajplung.2001.280.6.L1094 11350788

[70] Mestas J Hughes CC. Of mice and not men: differences between mouse and human immunology. J Immunol (2004) 172(5):2731–8. doi: 10.4049/jimmunol.172.5.2731 14978070

[71] Madonna S Girolomoni G Dinarello CA Albanesi C. The significance of IL-36 hyperactivation and IL-36R targeting in psoriasis. Int J Mol Sci (2019) 20(13):3318. doi: 10.3390/ijms20133318 31284527 PMC6650959

[72] Towne JE Renshaw BR Douangpanya J Lipsky BP Shen M Gabel CA. Interleukin-36 (IL-36) ligands require processing for full agonist (IL-36α, IL-36β, and IL-36γ) or antagonist (IL-36Ra) activity. J Biol Chem (2011) 286(49):42594–602. doi: 10.1074/jbc.M111.267922 PMC3234937 21965679

[73] D’Erme AM Wilsmann-Theis D Wagenpfeil J Holzel M Ferring-Schmitt S Sternberg S. IL-36gamma (IL-1F9) is a biomarker for psoriasis skin lesions. J Invest Dermatol (2015) 135(4):1025–32. doi: 10.1038/jid.2014.532 25525775

[74] Miura S Garcet S Salud-Gnilo C Gonzalez J Li X Murai-Yamamura M. IL-36 and IL-17A cooperatively induce a psoriasis-like gene expression response in human keratinocytes. J Invest Dermatol (2021) 141(8):2086–90. doi: 10.1016/j.jid.2021.01.019 33675789

[75] Carrier Y Ma HL Ramon HE Napierata L Small C O'Toole M. Inter-regulation of Th17 cytokines and the IL-36 cytokines in *vitro* and in *vivo*: implications in psoriasis pathogenesis. J Invest Dermatol (2011) 131(12):2428–37. doi: 10.1038/jid.2011.234 21881584

[76] Wilsmann-Theis D Schnell LM Ralser-Isselstein V Bieber T Schon MP Huffmeier U. Successful treatment with interleukin-17A antagonists of generalized pustular psoriasis in patients without IL36RN mutations. J Dermatol (2018) 45(7):850–4. doi: 10.1111/1346-8138.14318 29655177

[77] Plachouri KM Chourdakis V Georgiou S. The role of IL-17 and IL-17 receptor inhibitors in the management of generalized pustular psoriasis. Drugs Today (Barc) (2019) 55(9):587–93. doi: 10.1358/dot.2019.55.9.3020159 31584575

[78] Imafuku S Honma M Okubo Y Komine M Ohtsuki M Morita A. Efficacy and safety of secukinumab in patients with generalized pustular psoriasis: A 52-week analysis from phase III open-label multicenter Japanese study. J Dermatol (2016) 43(9):1011–7. doi: 10.1111/1346-8138.13306 26919410

[79] Saeki H Nakagawa H Nakajo K Ishii T Morisaki Y Aoki T. Efficacy and safety of ixekizumab treatment for Japanese patients with moderate to severe plaque psoriasis, erythrodermic psoriasis and generalized pustular psoriasis: Results from a 52-week, open-label, phase 3 study (UNCOVER-J). J Dermatol (2017) 44(4):355–62. doi: 10.1111/1346-8138.13622 PMC5412888 27726163

[80] Yamasaki K Nakagawa H Kubo Y Ootaki K Japanese Brodalumab Study G. Efficacy and safety of brodalumab in patients with generalized pustular psoriasis and psoriatic erythroderma: results from a 52-week, open-label study. Br J Dermatol Mar (2017) 176(3):741–51. doi: 10.1111/bjd.14702 27106510

[81] Catapano M Vergnano M Romano M Mahil SK Choon SE Burden AD. IL-36 promotes systemic IFN-I responses in severe forms of psoriasis. J Invest Dermatol Apr (2020) 140(4):816–26 e3. doi: 10.1016/j.jid.2019.08.444 PMC7097848 31539532

[82] Ganesan R Raymond EL Mennerich D Woska JR Caviness G Grimaldi C. Generation and functional characterization of anti-human and anti-mouse IL-36R antagonist monoclonal antibodies. MAbs (2017) 9(7):1143–54. doi: 10.1080/19420862.2017.1353853 PMC5627585 28726542

[83] Boehringer Ingelheim. European Commission approves SPEVIGO^®^ (spesolimab) for generalized pustular psoriasis flares (2022). Available at: https://www.boehringer-ingelheim.com/human-health/skin-diseases/gpp/european-commission-approves-spevigo-spesolimab-generalized (Accessed January 24, 2023).

[84] Bachelez H Choon SE Marrakchi S Burden AD Tsai TF Morita A. Inhibition of the interleukin-36 pathway for the treatment of generalized pustular psoriasis. N Engl J Med (2019) 380(10):981–3. doi: 10.1056/NEJMc1811317 30855749

[85] Bachelez H Choon SE Marrakchi S Burden AD Tsai TF Morita A. Trial of spesolimab for generalized pustular psoriasis. N Engl J Med (2021) 385(26):2431–40. doi: 10.1056/NEJMoa2111563 34936739

[86] Shao S Wang G. Commentary on a clinical trial of spesolimab, a humanized anti-interleukin-36 receptor monoclonal antibody, in generalized pustular psoriasis. Dermatol Ther (Heidelb) (2022) 12(12):2627–35. doi: 10.1007/s13555-022-00830-x PMC9674804 36208408

[87] Blair HA. Spesolimab: first approval. Drugs (2022) 82(17):1681–6. doi: 10.1007/s40265-022-01801-4 PMC9744699 36418672

[88] Burden AD Choon SE Gottlieb AB Navarini AA Warren RB. Clinical disease measures in generalized pustular psoriasis. Am J Clin Dermatol (2022) 23(Suppl 1):39–50. doi: 10.1007/s40257-021-00653-0 35061231 PMC8801406

[89] Farag A Visvanathan S Bachelez H Morita A Lebwohl M Barker JN. Spesolimab alters the molecular profile of lesional skin in patients with generalized pustular psoriasis with a clinical response (Abstract FC04). Br J Dermatol (Psoriasis: From Gene to Clinic 9th Int Congress; London UK; 9 to 11 December 2021) (2021) 186(1):34988979.

[90] Navarini AA Prinz JC Morita A Tsai TF Viguier MA Li L. Spesolimab improves patient-reported outcomes in patients with generalized pustular psoriasis: Results from the Effisayil 1 study. J Eur Acad Dermatol Venereol (2022) 37(4):730–6. doi: 10.1111/jdv.18820 36527385

[91] Morita A Tsai TF Yee EYW Okubo Y Imafuku S Zheng M. Efficacy and safety of spesolimab in Asian patients with a generalized pustular psoriasis flare: Results from the randomized, double-blind, placebo-controlled Effisayil 1 study. J Dermatol (2023) 50(2):183–94. doi: 10.1111/1346-8138.16609 PMC10092680 36282833

[92] Burden AD Okubo Y Zheng M. (Abstract 33007) Efficacy of spesolimab for the treatment of GPP flares across prespecified patient subgroups in the Effisayil 1 study. J Am Acad Dermatol (2022) 87(3):AB54. 10.1111/exd.14824 37140190

[93] Morita A Choon SE Bachelez H Anadkat MJ Marrakchi S Zheng M. Design of effisayil 2: A randomized, double-blind, placebo-controlled study of spesolimab in preventing flares in patients with generalized pustular psoriasis. Dermatol Ther (Heidelb) Jan (2023) 13(1):347–59. doi: 10.1007/s13555-022-00835-6 PMC9823166 36333618

[94] Morita A Strober B Burden AD Choon SE Anadkat MJ Marrakchi S. Efficacy and safety of subcutaneous spesolimab for the prevention of generalised pustular psoriasis flares (Effisayil 2): an international, multicentre, randomised, placebo-controlled trial. Lancet (2023) 402(10412):1541–51. doi: 10.1016/S0140-6736(23)01378-8 37738999

[95] Boehringer Ingelheim. Effisayil™ ON: A study to test long-term treatment with spesolimab in people with generalized pustular psoriasis who took part in a previous study . Available at: https://clinicaltrials.gov/ct2/show/record/NCT03886246 (Accessed January 04, 2023).

[96] Navarini AA Bachelez H Choon SE Burden AD Zheng M Morita A. (2023). Effisayil ON, an open-label, long-term extension study of spesolimab treatment in patients with generalized pustular psoriasis: interim results for flare treatment. J American Academy of Dermatology 89(3), AB44. doi: 10.1016/j.jaad.2023.07.178

[97] Farag A Visvanathan S Bachelez H Morita A Lebwohl M Barker JN. Spesolimab alters the molecular profile of lesional skin in patients with generalized pustular psoriasis with a clinical response (Abstract 103). In: 4th Inflammatory Skin Disease Summit (ISDS), New York, November 3-6, 2021. Available at: https://www.isds2021.org/wp-content/uploads/2019/06/ISDS-2021_Late-Breaking-Abstracts-1.pdf.

[98] AnaptysBio. (Trial protocol) A Single Arm Multiple Dose Study to Assess the Efficacy and Safety of ANB019 in Subjects with Generalized Pustular Psoriasis . Available at: https://clinicaltrials.gov/ProvidedDocs/02/NCT03619902/Prot_000.pdf (Accessed 14 April 2022).

[99] AnaptysBio. AnaptysBio presents updated data from imsidolimab phase 2 GALLOP trial in generalized pustular psoriasis . Available at: https://ir.anaptysbio.com/news-releases/news-release-details/anaptysbio-presents-updated-data-imsidolimab-phase-2-gallop (Accessed October 19, 2023).

[100] Warren RB Reich A Kaszuba A Placek W Griffiths CEM Zhou J. Imsidolimab, an anti-interleukin-36 receptor monoclonal antibody, for the treatment of generalized pustular psoriasis: results from the phase II GALLOP trial. Br J Dermatol (2023) 189(2):161–9. doi: 10.1093/bjd/ljad083 37120722

[101] Gudjonsson JE Randazzo B Zhou J. Imsidolimab in the treatment of adult subjects with generalized pustular psoriasis: Design of a pivotal phase 3 clinical trial and a long-term extension study. J American Academy of Dermatology (2022) 87(3):AB70. doi: 10.1016/j.jaad.2022.06.313

[102] Todorovic V Su Z Putman CB Kakavas SJ Salte KM McDonald HA. Small molecule IL-36gamma antagonist as a novel therapeutic approach for plaque psoriasis. Sci Rep (2019) 9(1):9089. doi: 10.1038/s41598-019-45626-w 31235749 PMC6591177

[103] Regeneron Pharmaceuticals Inc. Ascending dose study of the safety and tolerability of REGN6490 in healthy volunteers (NCT04616079) . Available at: https://clinicaltrials.gov/ct2/show/NCT04616079 (Accessed February 20, 2023).

[104] Regeneron Pharmaceuticals Inc. Study of the safety, tolerability, and pharmacokinetics of REGN6490 in healthy Japanese adult volunteers (NCT04616105). Available at: https://clinicaltrials.gov/ct2/show/NCT04616105. (Accessed February 20, 2023).

[105] Fujita H Gooderham M Romiti R. Diagnosis of generalized pustular psoriasis. Am J Clin Dermatol (2022) 23(Suppl 1):31–8. doi: 10.1007/s40257-021-00652-1 PMC8777178 35061226

[106] Song HS Kim SJ Park TI Jang YH Lee ES. Immunohistochemical comparison of IL-36 and the IL-23/th17 axis of generalized pustular psoriasis and acute generalized exanthematous pustulosis. Ann Dermatol (2016) 28(4):451–6. doi: 10.5021/ad.2016.28.4.451 PMC4969474 27489427

[107] Meier-Schiesser B Feldmeyer L Jankovic D Mellett M Satoh TK Yerly D. Culprit drugs induce specific IL-36 overexpression in acute generalized exanthematous pustulosis. J Invest Dermatol (2019) 139(4):848–58. doi: 10.1016/j.jid.2018.10.023 30395846

[108] Stadler PC Oschmann A Kerl-French K Maul JT Oppel EM Meier-Schiesser B. Acute generalized exanthematous pustulosis: clinical characteristics, pathogenesis, and management. Dermatol (2023) 239(3):328–33. doi: 10.1159/000529218 36702114

[109] Misiak-Galazka M Zozula J Rudnicka L. Palmoplantar pustulosis: recent advances in etiopathogenesis and emerging treatments. Am J Clin Dermatol (2020) 21(3):355–70. doi: 10.1007/s40257-020-00503-5 PMC7275027 32008176

[110] Burden AD Bissonnette R Navarini AA Murakami M Morita A Haeufel T. Spesolimab efficacy and safety in patients with moderate-to-severe palmoplantar pustulosis: A multicentre, double-blind, randomised, placebo-controlled, phase IIb, dose-finding study. Dermatol Ther (Heidelb) (2023) 13(10):2279–97. doi: 10.1007/s13555-023-01002-1 PMC10539230 37731086

[111] Strober B Kotowsky N Medeiros R Mackey RH Harrold LR Valdecantos WC. Unmet medical needs in the treatment and management of generalized pustular psoriasis flares: evidence from a survey of corrona registry dermatologists. Dermatol Ther (Heidelb) (2021) 11(2):529–41. doi: 10.1007/s13555-021-00493-0 PMC8018987 33638115

[112] Gooderham MJ Van Voorhees AS Lebwohl MG. An update on generalized pustular psoriasis. Expert Rev Clin Immunol (2019) 15(9):907–19. doi: 10.1080/1744666x.2019.1648209 31486687

[113] Strober B Leman J Mockenhaupt M Nakano de Melo J Nassar A Prajapati VH. Unmet educational needs and clinical practice gaps in the management of generalized pustular psoriasis: global perspectives from the front line. Dermatol Ther (Heidelb) (2022) 12(2):381–93. doi: 10.1007/s13555-021-00661-2 PMC8850517 34904208

[114] Puig L Choon SE Gottlieb AB Marrakchi S Prinz JC Romiti R. Generalized pustular psoriasis: a global Delphi consensus on clinical course, diagnosis, treatment goals, and disease management. J Eur Acad Dermatol Venereol (2023) 37(4):737–52. doi: 10.1111/jdv.18851 36606566

[115] Elias M Zhao S Le HT Wang J Neurath MF Neufert C. IL-36 in chronic inflammation and fibrosis - bridging the gap? J Clin Invest (2021) 131(2):e144336. doi: 10.1172/JCI144336 33463541 PMC7810483

[116] Manzanares-Meza LD Valle-Rios R Medina-Contreras O. Interleukin-1 receptor-like 2: one receptor, three agonists, and many implications. J Interferon Cytokine Res (2022) 42(2):49–61. doi: 10.1089/jir.2021.0173 35171706

[117] Russell SE Horan RM Stefanska AM Carey A Leon G Aguilera M. IL-36alpha expression is elevated in ulcerative colitis and promotes colonic inflammation. Mucosal Immunol (2016) 9(5):1193–204. doi: 10.1038/mi.2015.134 26813344

[118] Leon G Hussey S Walsh PT. The diverse roles of the IL-36 family in gastrointestinal inflammation and resolution. Inflammation Bowel Dis (2021) 27(3):440–50. doi: 10.1093/ibd/izaa232 32860042

[119] Gudjonsson JE Tsoi LC Ma F Billi AC van Straalen KR Vossen A. Contribution of plasma cells and B cells to hidradenitis suppurativa pathogenesis. JCI Insight (2020) 5(19):e139930. doi: 10.1172/jci.insight.139930 32853177 PMC7566715

[120] Zouboulis CC Frew JW Giamarellos-Bourboulis EJ Jemec GBE Del Marmol V Marzano AV. Target molecules for future hidradenitis suppurativa treatment. Exp Dermatol (2021) 30 Suppl 1:8–17. doi: 10.1111/exd.14338 34085329

[121] Boutet MA Nerviani A Lliso-Ribera G Lucchesi D Prediletto E Ghirardi GM. Interleukin-36 family dysregulation drives joint inflammation and therapy response in psoriatic arthritis. Rheumatol (Oxford) (2020) 59(4):828–38. doi: 10.1093/rheumatology/kez358 PMC7188345 31504934

[122] Frey S Derer A Messbacher ME Baeten DL Bugatti S Montecucco C. The novel cytokine interleukin-36alpha is expressed in psoriatic and rheumatoid arthritis synovium. Ann Rheum Dis (2013) 72(9):1569–74. doi: 10.1136/annrheumdis-2012-202264 23268368

[123] Li T Chubinskaya S Esposito A Jin X Tagliafierro L Loeser R. TGF-beta type 2 receptor-mediated modulation of the IL-36 family can be therapeutically targeted in osteoarthritis. Sci Transl Med (2019) 11(491):eaan2585. doi: 10.1126/scitranslmed.aan2585 31068441 PMC7102613

[124] Wang XR Xiao JP Wang DG. Elevated levels of serum IL-36alpha in patients with systemic lupus erythematosus. BioMed Rep (2021) 15(3):76. doi: 10.3892/br.2021.1452 34405048 PMC8330003

[125] Mai SZ Li CJ Xie XY Xiong H Xu M Zeng FQ. Increased serum IL-36alpha and IL-36gamma levels in patients with systemic lupus erythematosus: Association with disease activity and arthritis. Int Immunopharmacol (2018) 58:103–8. doi: 10.1016/j.intimp.2018.03.011 29571080

[126] Maronese CA Pimentel MA Li MM Genovese G Ortega-Loayza AG Marzano AV. Pyoderma gangrenosum: an updated literature review on established and emerging pharmacological treatments. Am J Clin Dermatol (2022) 23(5):615–34. doi: 10.1007/s40257-022-00699-8 PMC9464730 35606650

[127] Barbieux C Bonnet des Claustres M Fahrner M Petrova E Tsoi LC Gouin O. Netherton syndrome subtypes share IL-17/IL-36 signature with distinct IFN-α and allergic responses. J Allergy Clin Immunol (2022) 149(4):1358–72. doi: 10.1016/j.jaci.2021.08.024 34543653

[128] Alavi A Prens EP Kimball AB Krueger JG Mukhopadhyay S Wang H. Spesolimab for hidradenitis suppurativa: A proof-of-concept study. Journal of the American Academy of Dermatology (2023) 89(3):AB89. doi: 10.1016/j.jaad.2023.07.358

[129] Neurath MF. IL-36 in chronic inflammation and cancer. Cytokine Growth Factor Rev (2020) 55:70–9. doi: 10.1016/j.cytogfr.2020.06.006 32540133

[130] Chelvanambi M Weinstein AM Storkus WJ. IL-36 signaling in the tumor microenvironment. Adv Exp Med Biol (2020) 1240:95–110. doi: 10.1007/978-3-030-38315-2_8 32060891 PMC7043067

[131] Byrne J Baker K Houston A Brint E. IL-36 cytokines in inflammatory and Malignant diseases: not the new kid on the block anymore. Cell Mol Life Sci (2021) 78(17-8):6215–27. doi: 10.1007/s00018-021-03909-4 PMC8429149 34365521

